# Testosterone as a Biomarker for Quality of Life (QOL) Following Androgen Deprivation Therapy (ADT) and Stereotactic Body Radiotherapy (SBRT)

**DOI:** 10.7759/cureus.44440

**Published:** 2023-08-31

**Authors:** Sarthak Shah, Abigail Pepin, Matthew Forsthoefel, Jessica Burlile, Brian T Collins, Suy Simeng, Nima Aghdam, Sean Collins

**Affiliations:** 1 Radiation Medicine, MedStar Georgetown University Hospital, Washington, DC, USA; 2 Radiation Oncology, University of Pennsylvania Abramson Cancer Center, Philadelphia, USA; 3 Radiation Medicine, Mayo Clinic, Rochester, USA; 4 Radiation Medicine, Tampa General Hospital (TGH) Cancer Institute, Tampa, USA; 5 Radiation Oncology, Beth Israel Deaconess Medical Center, Harvard Medical School, Boston, USA

**Keywords:** minimally important difference, adt, testosterone, quality of life, epic, cyberknife, sbrt, prostate cancer

## Abstract

Background: Androgen deprivation therapy (ADT) causes fatigue and sexual dysfunction. The time to testosterone recovery depends on patient and treatment-specific characteristics. The kinetics of testosterone recovery in men treated with neoadjuvant ADT and stereotactic body radiotherapy (SBRT) is not well established. This study seeks to characterize testosterone recovery and evaluate its relationship with the improvement in patient-reported hormonal and sexual function.

Methods: Institutional review board (IRB) approval was obtained for retrospective review of prospectively collected data. All patients with localized prostate cancer treated with short-course ADT (3-6 months of Leuprolide) and robotic SBRT (35-36.25 Gy in five fractions) at a single institution were included in this analysis. Testosterone levels were measured at the start of radiation, every 3 months for the first year, and every 6 months thereafter. Total testosterone recovery was defined as a serum level of >230 ng/dL. Sexual and hormonal function was recorded using the Expanded Prostate Index Composite (EPIC)-26 prior to ADT initiation, the first day of SBRT, and at each follow-up. The EPIC-26 subdomain scores were transformed to a 0-100 scale with higher scores reflecting less bother.

Results: Between January 2009 and May 2018, 122 men with a median age of 72 years (range: 55-89 years) received ADT followed by SBRT. Thirty-two percent (N=39) were black and 27% [N=39 were obese (BMI > 30)]. The median pre-SBRT testosterone level was 15 ng/dL (range: 3-89 ng/dL). Around 77% (N=94) of patients received 3 months of ADT. The median pre-ADT EPIC-26 Hormone and Sexual Domain Scores were 94 and 41, respectively. At 12 months, 71% (N=87) of patients recovered to a eugonadal state with a mean recovery time of 4 months post-SBRT. Hormonal and sexual subdomain scores declined significantly following ADT but recovered to within the minimally important difference (MID) for sexual and hormonal domain scores by 12 months post-SBRT.

Conclusions: Testosterone recovery following short-course ADT with leuprolide and SBRT occurs rapidly in the majority of patients within one year after treatment. Quality of life domain improvements followed the testosterone recovery trend closely. Testosterone testing at follow-up appointments would allow for anticipatory counseling that may limit the bother associated with temporary quality of life decrements.

## Introduction

The safety of stereotactic body radiotherapy (SBRT) for the treatment of localized treatment of prostate cancer has been documented, and long-term follow-up suggests a high rate of cancer control [1-2]. Treatment is well-tolerated and toxicity is low even in elderly patients [3-4]. Testicular scatter results in small (10%-30%) transient testosterone declines that do not impact fatigue or sexual function [5-6].

Androgen deprivation therapy (ADT) is an important component of treatment for select patients with unfavorable intermediate and high-risk prostate cancer. ADT has been shown to decrease the risk of distant metastases in unfavorable intermediate-risk prostate cancer [7]. Multiple studies have shown improved overall survival in intermediate-risk patients with the addition of ADT when compared to radiation therapy (RT) alone [8]. GnRH agonists, such as leuprolide, induce a marked testosterone decrease in castrate levels over several weeks. These low testosterone levels are associated with well-established hypogonadal symptoms such as fatigue, depression, hot flashes, decreased libido, impotence, gynecomastia, and weight gain [9]. In fact, in one study, men reported a willingness to sacrifice a 9.5% absolute gain in prostate cancer-specific survival in order to be spared the effects of long-term vs short-term ADT [10]. Providing information about expected testosterone recovery (TR) (and subsequent functional recovery) is key to building trust and adhering to patients’ priorities and values.

The timeline of TR after short-term ADT varies, but most reports find that the majority of men recover normal testosterone levels within six months after ADT cessation [8, 11]. The time to TR is variable and depends on both patient and treatment-specific characteristics. Patient-related characteristics include age, race, and BMI. In general, TR is slower in older obese men and faster in black men [12]. As for treatment-related characteristics, the shorter the length of ADT administration the more rapid the TR [13]. Additionally, multiple studies have shown that hypofractionation is not associated with testosterone suppression [8].

A suitable biomarker for quality of life is elusive, in part because the facets of physical, mental, and sexual well-being are complex and difficult to measure. Evidence has shown that low testosterone levels can cause decreased function in all of the aforementioned domains [14]. Concurrent with this recovery in testosterone levels, patient-reported quality of life (QOL) scores also return to baseline after SBRT and ADT, often by one year [5]. In this article, we report on TR after neoadjuvant ADT and SBRT and propose testosterone as a potential biomarker for male QOL in prostate cancer patients. This article was previously presented as a meeting poster at the 2019 ASTRO Annual Meeting on September 1, 2019.

## Materials and methods

Patient selection

Patients eligible for inclusion in this study had histologically confirmed prostate cancer treated with a combination short-course ADT and SBRT. Approval from the MedStar Georgetown University Institutional Review Board was obtained for a retrospective review of data that was prospectively collected in our institutional database (IRB#: 2009-510).

Treatment planning and delivery

All patients received between three and six months of ADT consisting of the luteinizing hormone-releasing hormone agonist, leuprolide. In general, SBRT was delivered three months after the first injection in order to maximize prostate size reduction and minimize radiation dose to surrounding healthy tissues [15]. SBRT was delivered utilizing the CyberKnife robotic radiosurgical system (Accuray, Inc., Sunnyvale, CA, USA). Fiducial placement, treatment planning MRI, and CT simulation procedures have been previously described [16]. Briefly, the trans-perineal placement of four gold fiducials accommodated X-ray-guided prostate localization and beam adjustment. Fiducial separation and non-overlapping positioning permitted orthogonal imaging required for 6D tracking. The clinical target volume (CTV) was defined as the prostate and proximal seminal vesicles. The CTV was expanded 5 mm in all directions except 3 mm posteriorly to generate the planning target volume (PTV). Patients were treated to a prescription dose of 35-36.25 Gy, with a median dose of 36.25, to the PTV delivered in five fractions over two weeks based on treatment planning performed using Multiplan (Accuray Inc., Sunnyvale, CA, USA). Treatment beams that directly traversed the testis were blocked and the scatter dose was kept to a minimum [6]. The dose volume histogram goal for the penile bulb was V29 Gy < 3 cc [17]. 

Follow-up

Serum testosterone levels were obtained prior to the first SBRT treatment and during routine follow-up visits every three months for the first year and every six months thereafter. In general, serum samples were collected in the morning to limit the impact of circadian variance. Biochemical hypogonadism was defined as total serum testosterone levels below 230 ng/dL [18], and TR was defined as reaching a serum testosterone level of at least 230 ng/dL.

Sexual and hormonal function was assessed using the Expanded Prostate Index Composite (EPIC)-26 prior to ADT initiation, the first day of SBRT, and at each follow-up. The EPIC sexual function questionnaire asked patients to assess the following: ability to have an erection, ability to reach orgasm, the quality of erections, frequency of erections compared with the frequency of desired erections, and overall sexual function. The EPIC hormonal domain questions addressed hot flashes, breast tenderness or enlargement, feeling depressed, lack of energy, and change in body weight. To statistically compare changes in EPIC domain scores at each time point, for each EPIC question, the level of responses was assigned a score and transformed onto a 0-100 scale with lower scores reflecting worsening sexual or hormonal symptoms. The MID utilized for the hormonal and sexual domains were 10.6 and 9.0, respectively [19].

## Results

Between January 2009 and May 2018, 122 men were treated per our institutional SBRT protocol with a combination short course ADT. Patient characteristics are presented in Table 1. The observation period was from initial consult to 10 years follow-up, with a median follow-up of 12 months. The median age of all patients was 72 years (range, 55-89 years). Sixty-three patients (52%) were Caucasian and 39 patients were obese (27%). Sixty-three patients (52%) were intermediate-risk and 49 patients (40%) were high-risk per D’Amico’s risk group. Ninety-four patients received three months of ADT (77%).

**Table 1:** Patient characteristics and treatment. AUA, American Urological Baseline; SBRT, stereotactic body radiotherapy; BMI, body mass index

	Number of patients (N=122)	Percent of patients (%)
Age (years): Median 72 (54.49-88.32)		
60-69	8	6.40
70-79	49	40
>80	65	53
Race		
White	63	52
Black	39	32
Other	20	16
Prostate volume (cc)	Median 41.124 (7 - 160)	
BMI (kg/m²)		
<18.5	1	1
18.5-24.9	28	23
25.0-29.9	59	48
30.0-34.9	25	20
35.0-39.9	5	4
40.0-44.9	4	3
Charlson Comorbidity Index		
0	59	48
1	30	25
2	20	16
3	13	11
Risk Group (D’Amico)		
Low	10	7
Intermediate	63	52
High	49	40
Hormone Therapy		
3 months	94	77
4 months	10	8
6 months	18	15
SBRT Dose		
35	41	34
36.25	81	66
AUA Baseline		
0-7 (Mild)	47	39
8-19 (Moderate)	55	45
≥20 (Severe)	20	16

**Figure 1:**
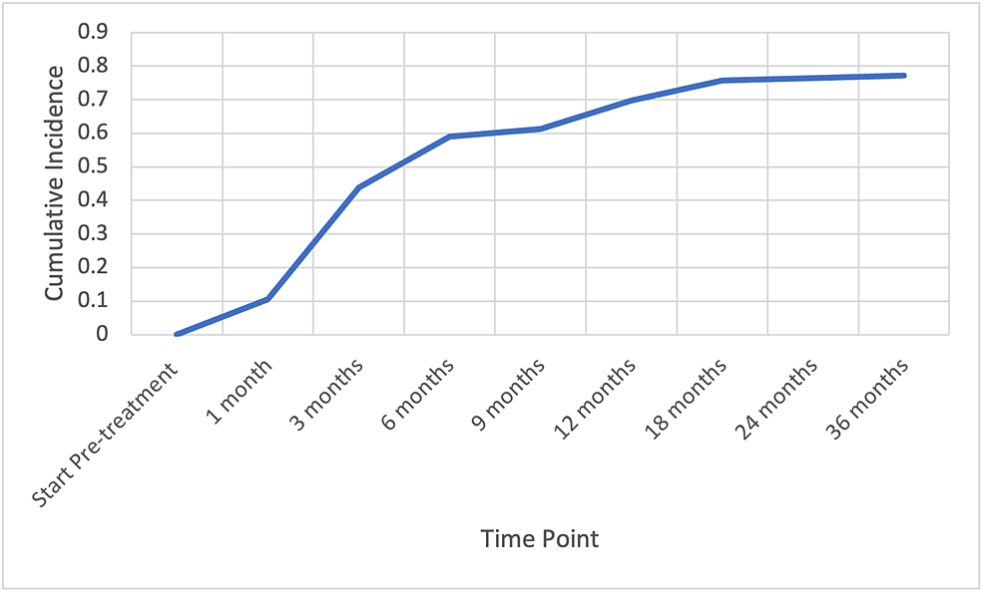
Cumulative incidence of patients recovering normal total testosterone levels (> 230 ng/dL) with time.

Median pre-radiotherapy testosterone level was 15 ng/dL (range, 3-89 ng/dL). At 12 months post-SBRT, 87 patients (71%) recovered to a eugonadal state with a mean time to recovery of 4 months. See Figure 1.

**Figure 2:**
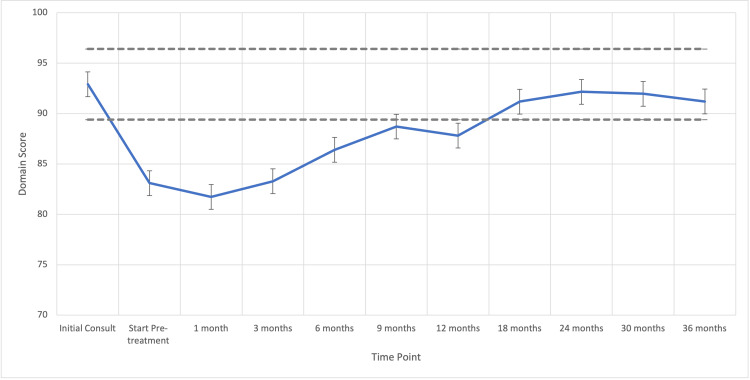
Hormonal QOL following ADT and SBRT for prostate cancer. Mean hormonal domain EPIC scores range from 0 to 100 with higher values representing a more favorable health-related QOL. Error bars indicate SEM. Dashed lines represent the calculated minimally important difference values. QOL, quality of life; ADT, androgen deprivation therapy; SBRT, stereotactic body radiotherapy; EPIC, Expanded Prostate Index Composite; SEM, standard error mean

**Figure 3:**
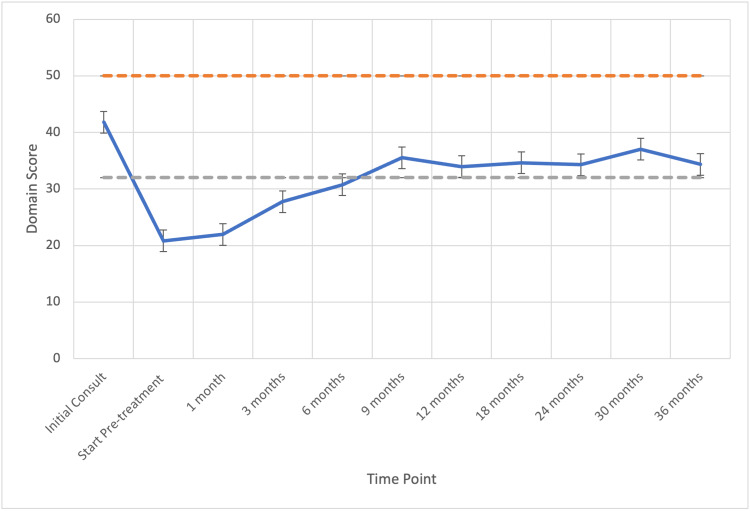
Sexual QOL following ADT and SBRT for prostate cancer. Mean sexual domain EPIC scores range from 0 to 100 with higher values representing a more favorable health-related QOL. Error bars indicate SEM. Dashed lines represent the calculated minimally important difference values. QOL, quality of life; ADT, androgen deprivation therapy; SBRT, stereotactic body radiotherapy; EPIC, Expanded Prostate Index Composite; SEM, standard error mean

The median pre-radiotherapy EPIC-26 Hormone and Sexual Domain Scores were 94 (range, 40-100) and 41 (range, 31-62), respectively. Hormonal and sexual bother subdomain scores declined significantly following radiation to 20 (MID = 10.6) and 88 (MID = 9.0) respectively, but recovered in 71% (N=86) and 57% (N=70) of the patients by 12 months post-SBRT, respectively (Figures 2-3). 

## Discussion

Low serum testosterone levels are a product of ADT and are associated with a slew of symptoms that adversely affect QOL in patients with prostate cancer. While on ADT, up to half of men report feeling less masculine, and up to 93% of men stop sexual activity (94% report a loss of sexual desire) [20]. The results we report from the present study show that ADT causes a significant acute decrease in both hormonal and sexual domain scores and that TR closely correlates with improvement in these same domains.

A marked decrease in testosterone occurs with the administration of GnRH agonists such as leuprolide. After ADT cessation, serum testosterone levels gradually return to normal in most men -- a cumulative incidence of recovery between 65.7% and >90% has been reported [11]. In our study, 71% of patients (N=86) recovered their testosterone by one year, and by 18 months after ADT cessation, TR reached a plateau where 76% (N=92) of patients had recovered to normal serum testosterone levels (Figure 1). Previous research has shown that the timeline of this recovery varies based on length and type of ADT, and can be prolonged because of patient factors such as older age, higher BMI, white race, lower testosterone nadir, and lower pre-treatment testosterone level [21].

Although the testosterone levels of most men did return to the normal range, about 23% of those in our study did not recover normal levels. This discrepancy suggests that these men may have been hypogonadal at baseline, which is an association that has been elucidated in other studies [21]. The relatively high median age (72 years) as well as low baseline EPIC-26 sexual domain score (39.5) indicate that it is probable that a sizeable portion of men was hypogonadal at baseline.

Ninety-four patients (77%) in our study received 3 months of ADT; the remainder received six. We found that serum testosterone levels recovered to normal a median of 4 months after ADT cessation. Other investigators have reported TR after short-course ADT ranging from 13 weeks to 2.1 years [8]. There is some evidence that shorter-acting GnRH agonists (1-month preparations vs. 3-month preparations) result in a faster return to normal testosterone levels, as well as a higher cumulative recovery [22]. A recent HERO Trial has also demonstrated Reguloix as an alternative for rapid testosterone recovery [23]. This may be an area for further research.

Previously published studies that have examined patient-reported QOL outcomes in conjunction with ADT and RT report poorer sexual and hormonal bother domain scores after initiation of ADT, but significant recovery over the course of 12-15 months [11]. Our study reports similar results, with an early drop in both EPIC hormonal and sexual domain scores beyond the threshold of a minimally important difference (MID). Regarding the sexual domain, men recover close to baseline within 6 months after the initiation of SBRT (approximately 9 months after the initiation of ADT, see Figure 2).

Previous research as well as our results in this study indicate that the restoration of normal serum testosterone levels correlates with restored sexual function and that the effects of short-term ADT are reversible. Others have shown that the long-term effectiveness of sexual aids (after TR) is not affected by the use of short-term ADT [24], and despite the differences in TR kinetics between patients treated with RT alone and RT plus ADT, patient-reported sexual function at one year appears similar [25]. Conversely, the gradual, long-term erectile decline that is observed years after ADT cessation should not be ascribed to ADT in the setting of normal testosterone levels, and instead should be attributed to radiation neurovascular effects and the normal aging process [5].

Our data show that patient-reported hormonal bother dips below the MID after initiation of SBRT/ADT but recovers within 6 months (Figure 3). In a publication using a single 3-month injection of ADT, patients reported hot flashes and sweats lasting a median of 13.6 months after ADT cessation -- the resolution of these symptoms closely correlated with TR. Additionally, nearly 31% of men in this small study reported the development of gynecomastia, even with a very short course of ADT [14]. In our study, overall hormonal domain scores recovered to above the threshold for MID at 6 months after initiation of SBRT.

Previous research has shown that men are willing to sacrifice some degree of prostate cancer-specific survival in order to be spared the effects of long-term ADT, in particular, the decline in sexual potency [10]. It is important to communicate with patients about the expected effects of both long-term and short-term ADT and discuss the expected functional recovery with each treatment. Sexual desire and erectile function have distinct patterns of change as serum testosterone levels are reduced [26]. A possible confounder is that radiation to the penile bulb can result in erectile dysfunction, conflicting with symptoms of hypogonadism [27]. Because testosterone levels are associated with sexual desire and sexual activity [28], and many men consider sexuality an important component of quality of life [10, 29], measuring testosterone levels periodically after ADT and SBRT can provide valuable information for patients and their partners. Return to normal testosterone levels may be particularly significant for men as studies have shown that once testosterone reaches a low-end-of-normal level, sexual function and feelings are frequently restored. As sexual performance is a complex and highly psychological phenomenon [30], knowing that recovering testosterone levels could be enough to improve a man’s sexual function.

The association between low testosterone levels, adverse side-effects from low levels, and the results of the present study demonstrating a strong correlation between testosterone levels and patient-reported QOL measures indicate that testosterone may be used as a biomarker for patient-reported QOL, especially in the hormonal and sexual domains as measured by EPIC-26.

Limitations of this study include its retrospective nature. Because of the observational nature of the study, data points were not available for all patients at every time point. This includes pre-ADT testosterone levels. As a result of baseline testosterone being unavailable, the proportion of baseline hypogonadal patients cannot be known.

## Conclusions

Stereotactic body radiotherapy and short-term ADT with leuprolide are proven to be safe and effective for treating localized prostate cancer. Declines in serum testosterone are striking but temporary, as most men recover levels to the normal range, although the timeline of recovery can vary based on the length of ADT and patient factors. Patient-reported QOL scores in the hormonal and sexual domains decline sharply, mirroring this decrease in testosterone, but return close to baseline as testosterone levels recover. Testosterone testing at follow-up appointments post-SBRT would allow for anticipatory counseling that may limit the bother associated with temporary QOL decrements.
